# Short-term variations in trabecular bone texture parameters associated to radio-clinical biomarkers improve the prediction of radiographic knee osteoarthritis progression

**DOI:** 10.1038/s41598-023-48016-5

**Published:** 2023-12-11

**Authors:** Ahmad Almhdie-Imjabbar, Hechmi Toumi, Eric Lespessailles

**Affiliations:** 1 Translational Medicine Research Platform, PRIMMO, University Hospital Centre of Orleans, Orleans, France; 2 Department of Rheumatology, University Hospital of Orleans, Orleans, France

**Keywords:** Predictive markers, Predictive markers, Osteoarthritis, Risk factors

## Abstract

The present study aims to examine whether the short-term variations in trabecular bone texture (TBT) parameters, combined with a targeted set of clinical and radiographic data, would improve the prediction of long-term radiographic knee osteoarthritis (KOA) progression. Longitudinal (baseline, 24 and 48-month) data, obtained from the Osteoarthritis Initiative cohort, were available for 1352 individuals, with preexisting OA (1 < Kellgren–Lawrence < 4) at baseline. KOA progression was defined as an increase in the medial joint space narrowing score from the 24-months to the 48-months control point. 16 regions of interest were automatically selected from each radiographic knee and analyzed using fractal dimension. Variations from baseline to 24 months in TBT descriptors as well as selected radiographic and clinical readings were calculated. Different logistic regression models were developed to evaluate the progression prediction performance when associating TBT variations with the selected clinical and radiographic readings. The most predictive model was mainly determined using the area under the receiver operating characteristic curve (AUC). The proposed prediction model including short-term variations in TBT parameters, associated with clinical covariates and radiographic scores, improved the capacity of predicting long-term radiographic KOA progression (AUC of 0.739), compared to models based solely on baseline values (AUC of 0.676, *p*-value < 0.008).

## Introduction

Osteoarthritis (OA) is among the leading causes of impaired mobility and chronic pain, affecting almost half of the population aged 65 years or older worldwide. OA is considered as the most prevalent form of arthritis^1, 2^. The knee is the primary joint of interest in OA imaging research^3^. An increased health care burden of knee Osteoarthritis (KOA) has been evidenced both in cross-sectional^4^ and in longitudinal^5^ studies with an annual global increase in the age standardized incidence rate based on the global burden of disease data over an almost three decade period. KOA is a musculoskeletal condition frequently encountered not only in primary care but also in orthopedic and rheumatology clinics. Reducing pain and decreasing the progression of joint damage in patients with KOA is still however a challenging task. Indeed, it has been elegantly demonstrated that cartilage thickness loss (− 0.1 mm) over 2 years was associated with only a 0.32 increase in WOMAC pain score^6^. Consequently, improvement in clinical symptoms may not be systematically associated with chondroprotective drugs. Furthermore, pain resolution is a complex clinical outcome, some recreational activities being associated with significant odds of pain resolution while others are not^7^.

The early detection and assessment of KOA prognostic factors are crucial for developing management and treatments that aim at preventing irreversible damage to the knee joint leading to arthroplasty. To assess the efficacy of new treatments aiming at reducing KOA structural progression is a difficult task because of the relatively low sensitivity and responsiveness of radiographic joint space width (JSW) for detecting disease progression^8^ in randomized clinical trials. Hence, there is a strong and growing interest in assessing whether, and under what conditions, other imaging biomarkers may be used to improve patient screening in phase III KOA trials^9, 10^.

Bone metabolism and particularly subchondral bone play a crucial role in the pathophysiology of KOA^11^. Knee alignment angle and loss of cartilage have been found to be associated with changes in both the microstructure and remodeling activity of the subchondral trabecular bone in KOA^12^. An overview of the interest of trabecular bone texture analysis (TBTA) in the assessment of KOA was recently published^9^. TBTA of subchondral bone on conventional knee radiographs was shown to be a promising method for identifying patients at-risk of KOA progression^10, 13–16^. Baseline TBT of the tibial plateau was found to be predictive of KOA progression in the well-phenotyped population of the OAI cohort^13^. In our previous studies^13, 17^, the best model included clinical (CLIN) covariates [age, gender, body mass index (BMI)], Kellgren–Lawrence (KL) grades, medial compartment joint space narrowing (JSNM) grades and TBT parameters. The proposed models were also evaluated with a more detailed set of clinical covariates by adding Western Ontario and McMaster University Osteoarthritis (WOMAC) pain scores^18^ with no gain in performance achieved^17^. Moreover, the performance of the TBT-based models was found invariant with respect to acquisition modality and image quality^17^ and robust to changing X-ray devices (centers)^13^.

The time-integrated values of radiographic trabecular bone texture (TBT) parameters associated with clinical and radiological descriptors have been previously used to evaluate the performance of KOA progression prediction models^10^. In this later case–control study, based on the Foundation for the National Institutes of Health (FNIH) dataset, a subgroup of the OAI cohort, the summed composite of 3 TBT parameters over 24 months improved the predictive ability with a statistically significant area under the receiver operating characteristic curve (AUC) of 0.649 over the use of the summed composite of 3 TBT parameters considering only baseline values (AUC of 0.635)^10^.

The novelty of the present original work includes the use of baseline to 24-month variations in the radiographic TBT parameters to develop novel prediction models of KOA progression over 4 years. In general, it is established that the variations of certain biomarkers make it possible to improve the prediction of the risk of occurrence of medical outcomes. For example, in osteoporosis trials, it was shown that changes in bone mass density can be considered as useful surrogate endpoints for fracture^19^.

The aim of this study was to evaluate the interest of using longitudinal changes in TBT parameters of the tibial subchondral plateau to predict KOA progression in the OAI database as compared to previous radiographic prediction models^13, 17^. For sensitivity analyses, the study also examined the performance of the models in predicting KOA progression defined as a radiographic joint space narrowing (JSN) in the medial compartment only as well as in any medial or lateral compartments. The prediction of these two outcomes was further assessed using the tibial subchondral TBT parameters of the medial regions of interest (ROIs) only, the lateral ROIs only or the central ROIs only, in addition to the use of TBT changes in the entire tibial subchondral plateau.

## Materials and methods

### Patients

This study included fully anonymized data obtained from the osteoarthritis initiative (OAI) cohort, in patients with KOA in at least one knee. The OAI is a longitudinal cohort study designed to identify biomarkers of the incidence and/or progression of KOA. In this cohort, both knees of 4796 participants were studied using bilateral posteroanterior fixed-flexion knee radiography with an annual follow-up over 8 years. The OAI study was performed in accordance with the relevant guidelines and regulations, and written and informed consent was obtained from participants prior to each clinical visit in the study. Details about the acquisition and grading protocols are available online at (https://nda.nih.gov/oai/study-details.html). Access to the raw data used in our study was approved by Osteoarthritis Initiative permission group of the National Institute of Mental Health Data Archive.

### Data selection

Exclusion criteria included participants about whom no clinical or radiographic information was available at baseline and at the 24-months visit, namely CLIN readings, WOMAC pain scores, KL grades, lateral joint space narrowing (JSNL) and JSNM grades. Participants with missing JSNM or JSNL grades at the 48-months visit were also excluded. As recommended by the European Medicines Agency^20^, only the knees with KL radiographic entry criteria of grades 2 or 3^13, 23^ were considered in the present study.

This inclusion/exclusion approach also included a quality control check in which it was verified whether the selected knee radiographs were suitable for TBT analysis. Radiographs showing materials (such as metallic materials, prostheses and screws), artifacts (unexpected vertical lines), over- or under-exposure in the subchondral zone, and radiographs not covering the complete subchondral area of interest, were also excluded.

It has been shown that variability estimates and statistical inferences are invalid if ignoring inter-eye correlation^21^. In the field of osteoarthritis, inter-knee JSW data were found to be highly positively correlated in different compartments^22^. In order to avoid such inter-knee correlation, the current study ensured the inclusion of only one knee per participant. Hence, if both knees fulfilled the aforementioned criteria, the most painful knee according to the participant’s WOMAC pain score was designated as index knee^23–28^. If both knees were equally painful, the one with a lower KL grade was excluded^23, 25, 26, 29^. In cases where both knees were still eligible, left knees^24, 28, 30^ were excluded.

### Definition of OA progression

OA progressors were defined as patients with KL grade 2 or 3 at baseline and with an increase in OARSI JSNM grade between the 24 and 48-months control points. Other patients were defined as non-progressors. Our main analysis focused on using the variations from baseline to 24 months of selected clinical and radiological descriptors for the prediction of KOA progression between 24 and 48 months. Patients with an increase in JSNM or JSNL grades from baseline to 24 months were therefore excluded.

KOA progressors in the OAI were primary medial and/or lateral compartment progressors. Consequently, sensitivity analyses were considered to address this fact by evaluating the proposed prediction models in four different scenarios, as described in Table 1.

**Table 1:** Definition of radiographic knee osteoarthritis (KOA) progression in 4 different scenarios.

Scenario	Definition of KOA progression	Number of knees studied	
		Total	Progressors
1	∆_V3V6_JSNM > 0 or ∆_V3V6_JSNL > 0	1352	164
2	∆_V3V6_JSNM > 0 and ∆_V3V6_JSNL = 0	1304	116
3	∆_V0V6_JSNM > 0 or ∆_V0V6_JSNL > 0	1352	176
4	∆_V0V6_JSNM > 0 and ∆_V0V6_JSNL = 0	1300	124

∆_V0V6_ represents the parameter’s variation from baseline to 48-months control point, whereas ∆_V3V6_ represents the parameter’s variation from 24-months control point to 48-months control point.

In scenario 1 (any progression), progressors were defined as patients with Δ_V3V6_JSNM or Δ_V3V6_JSNL > 0, where Δ_V3V6_ represents the parameter’s variation from the 24 to the 48-month control points. In scenario 2 (JSNM-only progression), KOA progression was defined as Δ_V3V6_JSNM > 0 mm and Δ_V3V6_JSNL = 0^17, 31, 32^. Consequently, in order to avoid misclassification of non-progressors, lateral compartment progressors (Δ_V3V6_JSNL > 0) were excluded.

For an inherent fairness in the comparison of prediction models based on 24-month variations in clinical and radiological values, progressors with (Δ_V0V3_JSNM > 0) or (Δ_V0V3_JSNL > 0) were excluded in all tested scenarios, where ∆_V0V3_ represents the parameter’s variation from baseline to 24 months.

Scenarios 3 and 4 are similar to scenarios 1 and 2, respectively. The only difference is that KOA progression is related to the increase in JSNM and/or JSNL grades from baseline to 48 months.

### Calculation of TBT parameters

**Figure 1:**
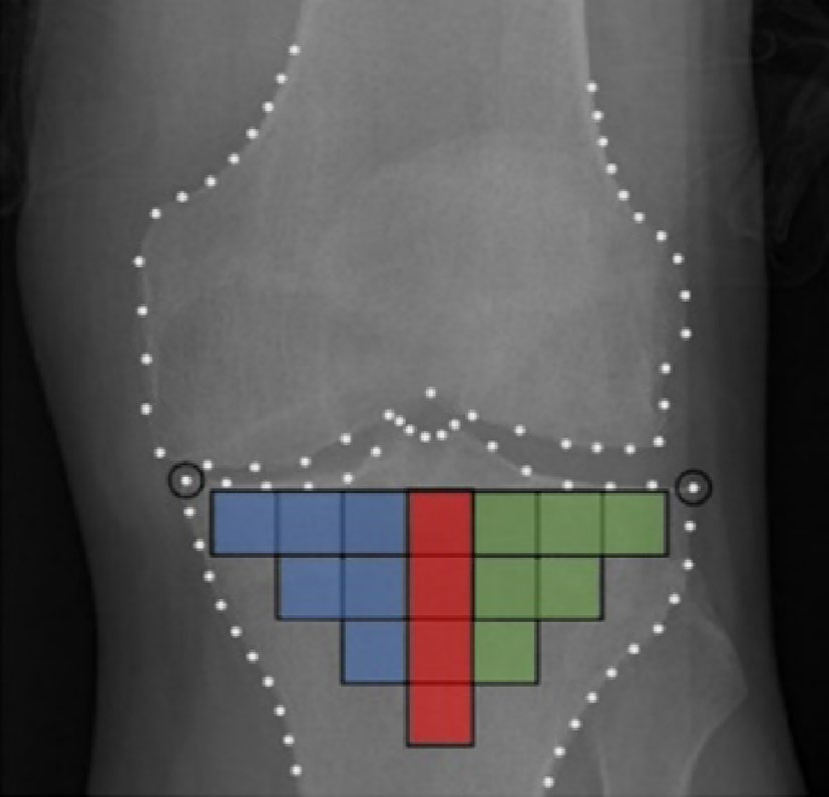
16 regions of interest (ROIs) automatically selected, covering the entire tibial subchondral bone structure. Medial, central and lateral ROIs are highlighted in blue, red and green, respectively.

The TBT of the respective radiographs was analyzed using fractal dimension, and the fractal parameters (*H*) were computed using the quadratic variations estimator (VAR)^33, 34^. As shown in Fig. 1, a total of squared-shape ROIs covering the entire tibial subchondral bone structure were automatically selected for each radiographic knee^13^, based on the lateral and medial extremities of the proximal tibia identified using the BoneFinder® software^35^. The TBT of each ROI was analyzed in both vertical and horizontal directions and in both microscopic and macroscopic scale, resulting in 64 descriptors for each knee. ROIs were not preprocessed before texture analysis.

### Longitudinal variations

Several clinical assessments and radiographic data measured at baseline, 24 months and 48 months were collected. Baseline to 24-month variations (∆) in TBT descriptors as well as in radiographic KL grades and clinical BMI and WOMAC pain readings were calculated using the arithmetic difference between the readings (*R*) at the 24-months visit (V3) and their corresponding readings at baseline.

1 $$\Delta R = R_{V3} - R_{Baseline}$$

### Statistical analysis

Logistic regression has been used for modelling KOA progression prediction^10, 13, 15^. Logistic regression is widely used in machine learning to predict the probability of occurrence of a binary event, using a logit function^36^. In this study, different logistic regression models were developed to evaluate the progression prediction performance when associating TBT variations over 24 months with the variations of a set of clinical and radiographic parameters, namely CLIN readings, WOMAC pain score, and KL grades.

In the present study, we evaluated the performance of 18 different prediction models. Models 1 to 5 were based on clinical covariates (CLIN) measured at baseline. Model 1 used CLIN covariates only. Model 2 used, in addition, WOMAC pain scores (CLINW). Since there was no improvement in the performance of Model 2 compared to Model 1, we decided not to include WOMAC pain scores in the other models evaluated. In models 3 and 4, the CLIN models were adjusted for KL and JSNM grades, respectively. Models 6–18 were based on the TBT descriptors, adjusted for CLIN, KL and JSNM, as well as their 24-month variations. We did not include JSNM variations to avoid the inherent correlation with the predefined outcome (Δ_V0V6_JSNM).

- Model 1: CLIN
- Model 2: CLINW
- Model 3: CLIN + KL
- Model 4: CLIN + JSNM
- Model 5: CLIN + KL + JSNM (Reference model)
- Model 6: TBT
- Model 7: TBT + CLIN + KL + JSNM
- Model 8: TBT + ∆CLIN + KL + JSNM
- Model 9: TBT + CLIN + ∆KL + JSNM
- Model 10: TBT + ∆CLIN + ∆KL + JSNM
- Model 11: ∆TBT + CLIN + KL + JSNM
- Model 12: ∆TBT + ∆CLIN + KL + JSNM
- Model 13: ∆TBT + CLIN + ∆KL + JSNM
- Model 14: ∆TBT + ∆CLIN + ∆KL + JSNM
- Model 15: TBT + ∆TBT + CLIN + KL + JSNM
- Model 16: TBT + ∆TBT + ∆CLIN + KL + JSNM
- Model 17: TBT + ∆TBT + CLIN + ∆KL + JSNM
- Model 18: TBT + ∆TBT + ∆CLIN + ∆KL + JSNM

To avoid overfitting, each model was evaluated using a tenfold cross-validation. The whole cross-validation process was then repeated 300 times. The average of the validation results was used as a single estimate of the model's predictive performance.

As a recommended and preferred metric for overall accuracy of machine learning algorithms, AUC was used to determine the most predictive model^37, 38^. The overall performance of a test is usually evaluated using the AUC representing the test’s sensitivity in function of its 1-specificity, (i.e. the false positive rate). Higher performance is obtained as the value approaches 1. Theoretically, an AUC of 0.5 means no discrimination and 0.7–0.8 is considered acceptable^38, 39^. Several widely-applied statistical metrics [balanced accuracy (BACC), positive predictive value (PPV), and negative predictive value (NPV)] were also calculated^40^. The BACC metric defined as the arithmetic mean of sensitivity and specificity, offers more reliable performance assessments for imbalanced data^41, 42^. Further details on the definition of these selected parameters are provided in our previous work^43^.

All statistical analyses were performed using the same procedure described in^17^, with newer versions for both the R Statistical tool (version 4.1.2, 2021-11-01) and the Modern Applied Statistics with S (MASS) package (version_7.3-55, 2022-01-12). As a recall, the stepwise Akaike Information Criterion (AIC) method^44^ of the MASS package was used to the most efficient parameter(s) before training the prediction models to limit overfitting effects. The method^45^ was also used to compare the Receiver Operating Characteristic (ROC) curves obtained by the different prediction models.

## Results

**Figure 2:**
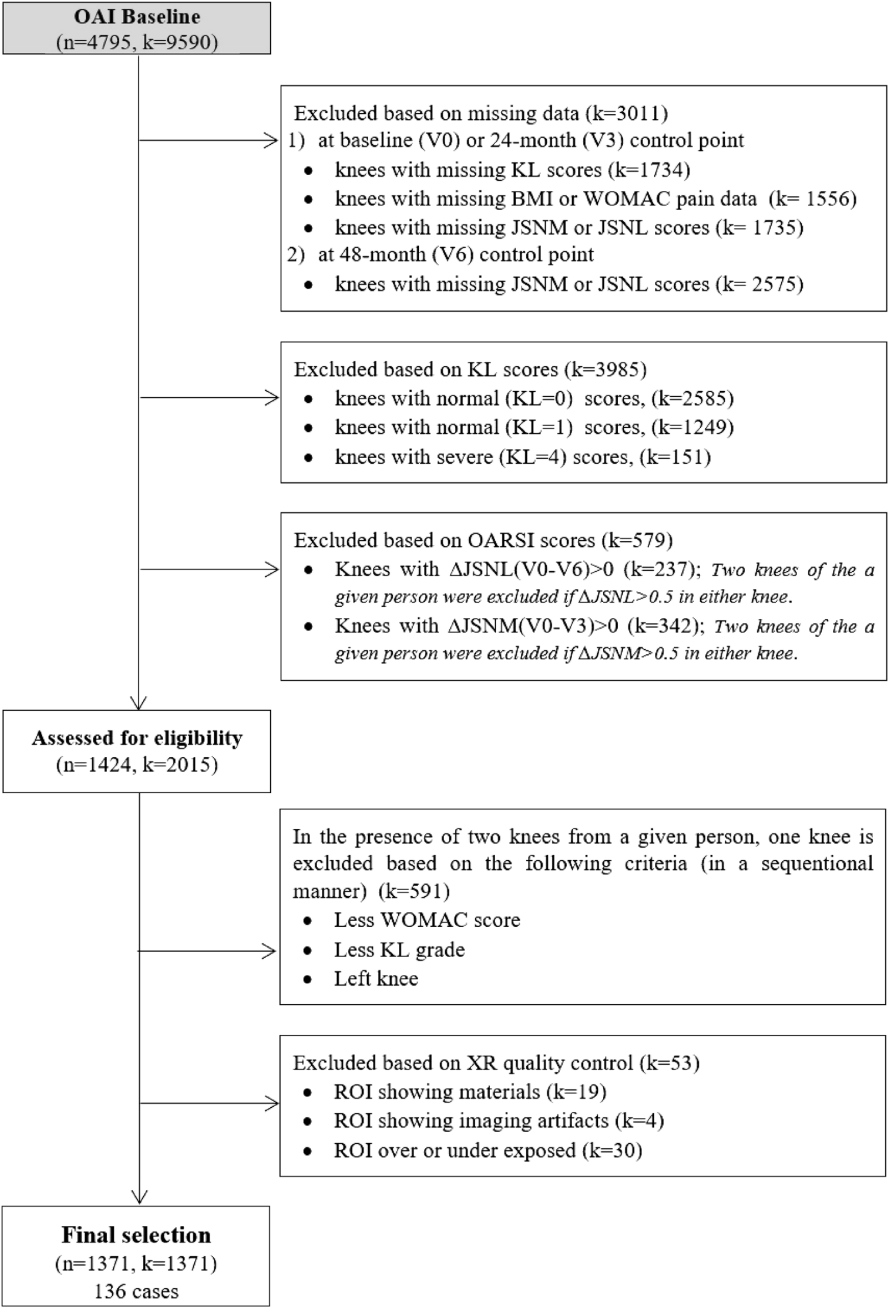
Consort diagram of the subject selection process (*n* is the number of patients and *k* is the corresponding number of knee radiographs at baseline).

Figure 2 illustrates the global inclusion/exclusion approach used in this study. 1352 knees (39% are left) from 1352 participants (58% are women) were considered eligible for this study. The number of progressors and non-progressors for each evaluated scenario is reported in Table 1.

The current study investigated the use of the variations between baseline and 24 months (V0V3) in TBT, clinical and radiological parameters to predict KOA progression between 24 and 48 months (V3V6). Using this definition of KOA progression, the results obtained by the different aforementioned models are reported in this paper for two scenarios.

Table 2 summarizes the performance results obtained by the aforementioned prediction models using TBT descriptors, calculated using the whole tibial subchondral zone.

**Table 2:** Performance results of scenario 1 and of scenario 2, using TBT analysis of the whole tibial subchondral zone (TBT).

Model N°	Models	BACC	PREC	PPV	NPV	AUC	pValue
Scenario 1							
1	CLIN	0.500	NaN	NaN	0.879	0.565 (0.521–0.610)	0.0654
2	CLINW	0.500	NaN	NaN	0.879	0.563 (0.518–0.608)	0.0443
3	CLIN ← KL	0.500	NaN	NaN	0.879	0.602 (0.557–0.647)	0.9945
4	CLIN ← JSNM	0.500	NaN	NaN	0.879	0.562 (0.517–0.608)	0.0477
5	CLIN ← KL + JSNM*	0.500	NaN	NaN	0.879	0.602 (0.556–0.648)	-
6	TBT	0.499	0.006	0.006	0.878	0.607 (0.563–0.652)	0.8574
7	TBT ← CLIN + KL + JSNM	0.499	0.028	0.028	0.878	0.636 (0.593–0.680)	0.0991
15	TBT + ∆TBT ← CLIN + KL + JSNM	0.509	0.221	0.221	0.881	**0.666** (0.623–0.709)	0.0143
Scenario 2							
1	CLIN	0.500	NaN	NaN	0.911	0.603 (0.550–0.655)	0.0009
2	CLINW	0.500	NaN	NaN	0.911	0.596 (0.544–0.649)	0.0003
3	CLIN ← KL	0.500	NaN	NaN	0.911	0.630 (0.578–0.683)	0.0052
4	CLIN ← JSNM	0.500	NaN	NaN	0.911	0.672 (0.623–0.722)	0.4746
5	CLIN ← KL + JSNM*	0.500	NaN	NaN	0.911	0.676 (0.627–0.725)	-
6	TBT	0.505	0.311	0.311	0.912	0.674 (0.623–0.725)	0.9442
7	TBT ← CLIN + KL + JSNM	0.506	0.247	0.247	0.912	0.704 (0.656–0.753)	0.1842
15	TBT + ∆TBT ← CLIN + KL + JSNM	0.534	0.381	0.381	0.917	**0.739** (0.695–0.782)	0.0076

BACC, PPV and NPV refer to balanced accuracy, positive predictive value and negative predictive value.

*refers to the reference model, and NaN refers to a Not-a-Number value where the sensitivity value was equal to zero. The model with the descriptor on the left of (←) is adjusted for the descriptor(s) on the right of (←). The highest AUC value obtained is bolded. In Scenario 1, progressors were defined as knees with 1 < KL < 4 at baseline and with an increase in JSNM or JSNL from 24 to 48 months. In Scenario 2, progressors were defined as knees with 1 < KL < 4 at baseline and with an increase in JSNM only from baseline to 48 months.

In Scenario 1, Model 15 combining baseline TBT descriptors and their variations over 24 months, adjusted for baseline CLIN, KL and JSNM readings achieved an AUC of 0.666, 95% Confidence Interval [CI]: 0.623, 0.709, outperforming a reference prediction model (Model 5) based exclusively on baseline CLIN readings adjusted for KL and JSNM which achieved an AUC of 0.602 (95% CI 0.556–0.648). The ROC curves of these models were statistically significantly different (*p*-value = 0.014). This improvement was mainly related to variations in TBT descriptors, although they could both be considered poorly predictive (0.6 < AUC < 0.7), Table 2. Including baseline and 24-month variation of TBT descriptors provided higher PPV and NPV results, notably with NPV values > 90% (Table 2).

In Scenario 2, in agreement with the previously observed results of Scenario 1, Model 15 achieved the highest AUC value (AUC = 0.739, 95% CI 0.695, 0.782), outperforming the reference model (Model 5) which achieved an AUC of 0.676 (95% CI 0.672–0.725), with a statistically significant difference between the AUCs of the two models (*p*-value = 0.008). The AUCs of the models including baseline and 24-month variation of TBT descriptors could be considered acceptable (0.7 < AUC < 0.8), compared to those obtained by the models excluding TBT descriptors (AUC < 0.7), (Table 2). Moreover, introducing the variations of CLIN and/or KL did not improve the performance results of Model 15, as seen in Tables S1 and S2 of Supplementary File 1. Hence, Model 15 was considered as the retained model.

In addition to the use of TBT changes in the entire tibial subchondral plateau, sensitivity analyses were conducted to address the impact of considering ROIs of only the medial (TBTM), lateral (TBTL) or central (TBTC) compartment of the tibia. See Fig. 1 for a visual representation of these three compartments. Results are reported in Table 3. The retained model performed better using the TBT descriptors of the whole tibial subchondral ROIs, with an AUC of 0.666, than using only TBTM, TBTL or TBTC descriptors with AUCs of 0.630, 0.656 and 0.632, respectively, for the progression of JSNM or JSNL (Scenario 1) and AUCs of 0.695, 0.704 and 0.675, respectively, compared to an AUC of 0.739 obtained by the retained model, for the progression of JSNM only (Scenario 2).

**Table 3:** Performance results of scenario 1 and of scenario 2, using TBT analysis of the whole tibial subchondral zone (TBT), the medial compartment only (TBTM), the lateral compartment only (TBTL) or the central compartment only (TBTC).

Models	BACC	PREC	PPV	NPV	AUC	*p***-value**
Scenario 1						
TBT + ∆TBT ← CLIN + KL + JSNM*	0.509	0.221	0.221	0.881	**0.666** (0.623–0.709)	–
TBTM + ∆TBTM ← CLIN + KL + JSNM	0.502	0.331	0.331	0.879	0.630 (0.585–0.675)	0.094
TBTL + ∆TBTL ← CLIN + KL + JSNM	0.503	0.235	0.253	0.879	0.659 (0.614–0.703)	0.680
TBTC + ∆TBTC ← CLIN + KL + JSNM	0.501	0.698	0.698	0.879	0.632 (0.587–0.677)	0.148
Scenario 2						
TBT + ∆TBT ← CLIN + KL + JSNM*	0.534	0.381	0.381	0.917	**0.739** (0.695–0.782)	–
TBTM + ∆TBTM ← CLIN + KL + JSNM	0.503	0.194	0.194	0.911	0.695 (0.652–0.739)	0.037
TBTL + ∆TBTL ← CLIN + KL + JSNM	0.521	0.529	0.529	0.914	0.704 (0.657–0.752)	0.073
TBTC + ∆TBTC ← CLIN + KL + JSNM	0.500	0.000	0.000	0.911	0.675 (0.625–0.725)	0.006

BACC, PPV and NPV refer to balanced accuracy, positive predictive value and negative predictive value.

*refers to the retained model. The model with the descriptor on the left of (←) is adjusted for the descriptor(s) on the right of (←). The highest AUC value obtained is bolded. In Scenario 1, progressors were defined as knees with 1 < KL < 4 at baseline and with an increase in JSNM or JSNL from 24 to 48 months. In Scenario 2, progressors were defined as knees with 1 < KL < 4 at baseline and with an increase in JSNM only from baseline to 48 months.

The same observation was found when considering the progression from baseline to 48 months (Scenarios 3 and 4), as seen in Tables S3 and S4 of Supplementary File 1. The retained model provided the highest AUC values when using TBT parameters of the whole tibial subchondral ROIs.

**Figure 3:**
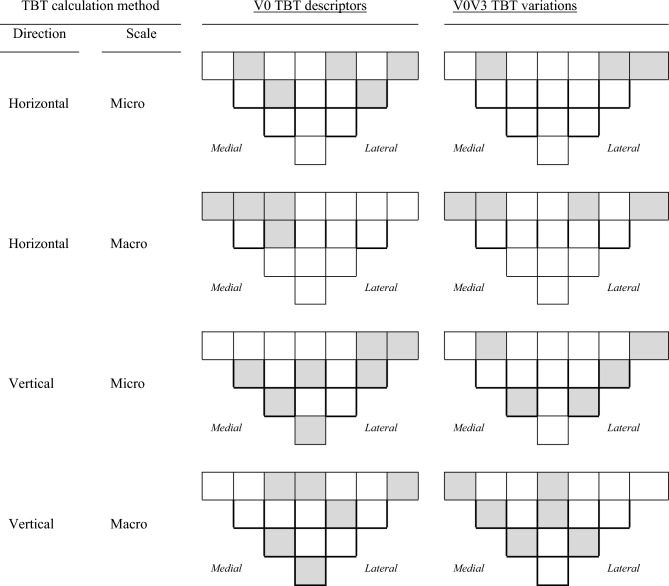
The most efficient baseline (V0) TBT descriptors (left) and baseline to 24-months (V0V3) TBT variations (right) chosen by the AIC algorithm for Scenario 2 (prediction of JSNM progression using the whole tibial subchondral regions of interest).

In order to better explain the impact of the variation of each TBT parameter (analyzed at micro and milli scales and in horizontal and vertical directions) on the performance of the prediction models, we show in Fig. 3 the ROIs automatically selected by the AIC-based optimization, highlighted by gray rectangles. Regarding the TBT analyses in the vertical direction, more ROIs were selected by the AIC method, compared to those selected in the horizontal direction. Regarding the TBT analyses in the horizontal direction, the number of ROIs selected by the AIC method from medial and lateral compartments was similar. However, no TBT parameters were selected from the central ROIs (Fig. 3).

Due to the limited number of progressors related to KOA progression of JSNL only (48 progressors vs. 1304 non-progressors) or progression of JSNL and JSNM together (1 progressor vs. 1351 non-progressors), these scenarios were not evaluated.

## Discussion

The originality of this paper was the use of short-term variations in TBT parameters associated with several clinical covariates (BMI, WOMAC pain) and radiological scores (KL) to improve the prediction of KOA progression.

This research demonstrated that based on baseline and further on 24-month longitudinal changes in TBT assessed on standardized plain knee radiographs, it was feasible to improve the prediction of KOA progression from 24 to 48 months in patients with mild KOA (knees with 2 ≤ KL ≤ 3) at baseline.

The use of TBT parameters at baseline and over 12 and 24 months (time integrated values) was previously evaluated^10^ as a predictor of 48-month radiographic and pain progression, in the FNIH cohort (600 knees), which is a sub-dataset of the OAI cohort. In the latter study, TBT parameters were extracted from a region of interest in the medial subchondral tibial region by fractal dimensions computed using the fractal signature analysis (FSA) method. The prediction model was evaluated using the TBT parameters extracted at baseline as well as at 12 months and 24 months, adjusted for baseline CLIN, WOMAC pain scores, race and joint space width. Introducing TBT parameters at baseline and time-integrated values over 12 months or 24 months, provided a gain in KOA prediction (AUC = 0.635, 0.633 and 0.649, respectively) compared to the use of covariates alone (AUC = 0.608)^10^. While the authors investigated the use of a composite score of 3 TBT parameters (horizontal filter intercept, horizontal filter quadratic slope and vertical filter quadratic slope) at each control point (baseline, 12 months and 24 months), they did not investigate the use of a composite score of TBT parameters at baseline associated to the variation of TBT parameters at 12- or 24 month control points. In addition, they only investigated TBT parameters of the medial compartment, while it has been demonstrated that regions from both lateral and medial compartments influence the performance of KOA progression prediction models^13^.

In the present study, the TBT variations from baseline TBT to the 24-month control point were evaluated in a larger dataset (1352 knees), taken from the whole OAI cohort. In addition, the TBT parameters were extracted from ROIs covering the entire subchondral tibial region. Since the definition of radiographic progression differs from that used in^10^.

The reason why the prediction of KOA progression was stronger when longitudinal changes of TBT parameters are taken into account rather than their baseline values alone is potentially because the degradation of subchondral bone tissue and its associated genes, variants and signaling pathways are more relevant to KOA progression than those implicated in OA onset. The assessment of pain scores showed no improvement on the reference model, and hence no link between pain and the progression of the disease can be suspected. In agreement with previous studies^9, 17^, the results of the present study showed that introducing baseline WOMAC pain values or variations in WOMAC pain between baseline and 24 months did not improve the performance of the prediction models, compared to using solely classical clinical covariates.

In line with the observation discussed by Janvier et al.^34^, the OA process involves changes in the actual topology of the microarchitecture of the horizontal bone trabeculae (characterized by vertical fractal analysis) in the late stages of KOA progression. These changes were observed at both the micro and milli scales of analysis^34^. The higher impact of micro or milli scale changes for JSNM progression prediction tends to indicate that the decrease in the joint space width is linked to the organization of the horizontal trabeculae. In the horizontal direction, the ROIs close to the articular cartilage play an important role in the performance of the prediction models. Radiographic KOA progression was influenced by TBT parameters from all ROIs (Table 3). This is true not only for the KOA progression in any compartment (JSNM or JSNL) but also for the KOA progression in the medial compartment uniquely, indicating that the complex mechanism associated with KOA progression depends on the entire tibial subchondral bone area.

The improvement in the prediction performance was mainly related to the variations of TBT descriptors (Table 2). For example, considering the AUC values obtained by Model 15 combining baseline TBT values with their variations over 24 months, the prediction capacity can be considered acceptable and was significantly higher (AUC of 0.739, *p*-value < 0.01) compared to other models excluding TBT descriptors, for which the performance varies from non-predictive to poorly predictive (AUC < 0.7).

Our study has a number of strengths. We used standardized clinical radiographs from the well-validated OAI cohort. We are the first to investigate the performance of progression prediction models using longitudinal variations of TBT parameters on such a large dataset. The real knowledge about texture parameters highlights the clinical relevance of our proposed model^9^. It makes sense to find a link between the variation in the microarchitecture of the subchondral bone and the evolution of the disease.

In this study, only one knee per participant was included to avoid any possible inter-knee correlation. Furthermore, knees with JSNM or JSNL progression from baseline to 24 months were excluded since the proposed prediction models involved the use of the variations of radiographic parameters within the same period of time.

The limitations of this study include the absence of femoral region of interest in our TBT analysis. The femoral subchondral bone might also provide additional information^46^. Another limitation is the lack of investigation of the association between radiographic KOA progression and 3D MRI bone texture^47^ or shape^48^. Integrating such parameters in our model is worth assessing. Recently, the combination of XR-based and MRI-based biomarkers, such as cartilage WORMS scores, have also been assessed for the prediction of KOA progression^32, 49, 50^. Nonetheless, given that radiography is less expensive and simpler to integrate into primary care practices, radiographic biomarkers might have stronger potential in KOA progression risk screening than MRI-based biomarkers^9^. It might also be interesting to compare the results of the prediction methods to those obtained by other machine learning methods such as the random forest^51^. Although radiographs have also been analyzed using DL approaches, we focused in our study on the use of a fractal-based TBT approach. While in DL-based methods more attention should be paid to the dissimilarity in image quality during the training process^52^, the TBT-based method used in our study has been demonstrated to be invariant to image quality and type.

Lastly, to test its consistency, the proposed model should be evaluated in a different cohort.

In this work, we studied the prediction of the radiographic progression (i.e., increase in JSNM or JSNL) as an endpoint. Even if joint space narrowing has been investigated as the primary outcome in most DMOAD trials^53^, and in KOA progression prediction models^9^, it would be of interest to evaluate the proposed models for the prediction of radio-clinical progression (i.e. lack of improvement in WOMAC pain in addition to radiographic progression), as considered by the FNIH^10^.

Finally, due to our selection criteria, we included only 1352 knees from the 9592 knees available at baseline in the OAI cohort. The OARSI clinical trials recommendations^54^ indicated the need to evaluate the ability of statistical models to predict the radiographic progression in subjects with definite radiographic change. We therefore limited our selection to knees with 2 ≤ KL ≤ 3, which was also the recommendation of the European Medicines Agency^20^ for studies of structure-modifying drugs.

There is a strong and growing interest in assessing whether, and under what conditions, imaging biomarkers may be used to improve patient screening in phase III trials for KOA^9, 55^. Our model predicted false progressors with great precision, an important issue from a clinical point of view, since their inclusion in a disease-modifying osteoarthritis-drug (DMOAD) randomized clinical trial would be counterproductive.

## Conclusions

Using the best available radio-clinical evidence in the prediction of knee osteoarthritis progression is essential in order to optimize the selection of most-at-risk patients for KOA progression.

In this study, a large set of radiomics were extracted using both baseline and short-term TBT variations from baseline to 24 months of KOA XRs. The results obtained showed that these radiomic descriptors are valuable imaging biomarkers for the prediction of 48-month KOA progression (AUC = 0.739).

### Supplementary Information

Supplementary Tables.

## Data Availability

The data that support the findings of this study are available from the OsteoArthritis Initiative (https://nda.nih.gov/oai/), but restrictions apply to the availability of these data, which were used under license for the current study, and so are not fully publicly available. Data are however available from the authors upon reasonable request and with permission of the OsteoArthritis Initiative.
