# Supplementary material for: Short-term variations in trabecular bone texture parameters associated to radio-clinical biomarkers improve the prediction of radiographic knee osteoarthritis progression
Source: Sci Rep. 2023 Dec 11;13:21952. doi: 10.1038/s41598-023-48016-5 (PMC10713565; doi:10.1038/s41598-023-48016-5)
Supplement: Supplementary file 1 — Supplementary Tables. [file 41598_2023_48016_MOESM1_ESM.docx]

**Supplementary file**

Table S1: Scenario 1 performance results of selected prediction models using TBT analysis of the whole tibial subchondral zone

| **Ref.** | **Model** | **BACC** | **PPV** | **NPV** | **AP** | **AUC (CI 95%)** | **pValue** |
| --- | --- | --- | --- | --- | --- | --- | --- |
| 1 | CLIN | 0.500 | NaN | 0.879 | 0,144 | 0.565 (0.521 – 0.610) | 0.0654 |
| 2 | CLINW | 0.500 | NaN | 0.879 | NaN | 0.563 (0.518 – 0.608) | 0.0443 |
| 3 | CLIN🡨KL | 0.500 | NaN | 0.879 | 0,153 | 0.602 (0.557 – 0.647) | 0.9945 |
| 4 | CLIN🡨JSNM | 0.500 | NaN | 0.879 | 0,140 | 0.562 (0.517 – 0.608) | 0.0477 |
| 5 | CLIN🡨KL+JSNM* | 0.500 | NaN | 0.879 | 0,158 | 0.602 (0.556 – 0.648) | 1.0000 |
| 6 | TBT | 0.499 | 0.006 | 0.878 | 0,171 | 0.607 (0.563 – 0.652) | 0.8574 |
| 7 | TBT🡨CLIN+KL+JSNM | 0.499 | 0.028 | 0.878 | 0,176 | 0.636 (0.593 – 0.680) | 0.0991 |
| 8 | TBT🡨∆CLIN+KL+JSNM | 0.499 | 0.089 | 0.879 | 0,172 | 0.619 (0.575 - 0.663) | 0.4830 |
| 9 | TBT🡨CLIN+∆KL+JSNM | 0.499 | 0.001 | 0.878 | 0,173 | 0.621 (0.576 - 0.666) | 0.4756 |
| 10 | TBT🡨∆CLIN+∆KL+JSNM | 0.498 | 0.002 | 0.878 | 0,168 | 0.605 (0.560 - 0.650) | 0.9025 |
| 11 | ∆TBT🡨CLIN+KL+JSNM | 0.501 | 0.551 | 0.879 | 0,192 | 0.632 (0.585 - 0.679) | 0.879 |
| 12 | ∆TBT🡨∆CLIN+KL+JSNM | 0.501 | 1.000 | 0.879 | 0,193 | 0.613 (0.565 - 0.662) | 0.879 |
| 13 | ∆TBT🡨CLIN+∆KL+JSNM | 0.503 | 0.629 | 0.879 | 0,170 | 0.608 (0.563 - 0.652) | 0.879 |
| 14 | ∆TBT🡨∆CLIN+∆KL+JSNM | 0.501 | 0.989 | 0.879 | 0,170 | 0.584 (0.538 - 0.629) | 0.879 |
| 15 | TBT+∆TBT🡨CLIN+KL+JSNM**^#^** | 0.509 | 0.221 | 0.881 | 0,204 | 0.**666** (0.623 – 0.709) | 0.0143 |
| 16 | TBT+∆TBT🡨∆CLIN+KL+JSNM | 0.513 | 0.275 | 0.882 | 0,202 | 0.652 (0.608 - 0.696) | 0.0809 |
| 17 | TBT+∆TBT🡨CLIN+∆KL+JSNM | 0.508 | 0.220 | 0.880 | 0,201 | 0.661 (0.618 - 0.704) | 0.0370 |
| 18 | TBT+∆TBT🡨∆CLIN+∆KL+JSNM | 0.511 | 0.259 | 0.881 | 0,199 | 0.644 (0.601 - 0.688) | 0.1707 |

BACC, PPV,NPV and AP refer to balanced accuracy, positive predictive value, negative predictive value and average precision. * refers to the reference model and # refers to the retained model. NaN refers to a Not-a-Number value where the sensitivity value was equal to zero. The model with the descriptor on the left of (🡨) is adjusted for the descriptor(s) on the right of (🡨). The highest AUC value obtained is bolded. In Scenario 1, progressors were defined as knees with 1<KL<4 at baseline and with a variation of > 0 from 24 months to 48 months in JSNM or JSNL.

Table S2: Scenario 2 performance results of selected prediction models using TBT analysis of the whole tibial subchondral zone

| **Ref.** | **Model** | **BACC** | **PPV** | **NPV** | **AP** | **AUC (CI 95%)** | **pValue** |
| --- | --- | --- | --- | --- | --- | --- | --- |
| 1 | CLIN | 0.500 | NaN | 0.911 | 0,119 | 0.603 (0.550 - 0.655) | 0.0009 |
| 2 | CLINW | 0.500 | NaN | 0.911 | NaN | 0.596 (0.544 - 0.649) | 0.0003 |
| 3 | CLIN🡨KL | 0.500 | NaN | 0.911 | 0,127 | 0.630 (0.578 - 0.683) | 0.0052 |
| 4 | CLIN🡨JSNM | 0.500 | NaN | 0.911 | 0,143 | 0.672 (0.623 - 0.722) | 0.4746 |
| 5 | CLIN🡨KL+JSNM* | 0.500 | NaN | 0.911 | 0,145 | 0.676 (0.627 - 0.725) | 1.0000 |
| 6 | TBT | 0.505 | 0.311 | 0.912 | 0,163 | 0.674 (0.623 - 0.725) | 0.9442 |
| 7 | TBT🡨CLIN+KL+JSNM | 0.506 | 0.247 | 0.912 | 0,174 | 0.704 (0.656 - 0.753) | 0.1842 |
| 8 | TBT🡨∆CLIN+KL+JSNM | 0.509 | 0.425 | 0.913 | 0,166 | 0.684 (0.637 - 0.732) | 0.7416 |
| 9 | TBT🡨CLIN+∆KL+JSNM | 0.508 | 0.316 | 0.912 | 0,178 | 0.702 (0.652 - 0.752) | 0.2532 |
| 10 | TBT🡨∆CLIN+∆KL+JSNM | 0.510 | 0.508 | 0.913 | 0,173 | 0.682 (0.633 - 0.731) | 0.8174 |
| 11 | ∆TBT🡨CLIN+KL+JSNM | 0.502 | 0.136 | 0.911 | 0,174 | 0.702 (0.653 - 0.750) | 0.2485 |
| 12 | ∆TBT🡨∆CLIN+KL+JSNM | 0.500 | 0.073 | 0.911 | 0,171 | 0.684 (0.633 - 0.735) | 0.7772 |
| 13 | ∆TBT🡨CLIN+∆KL+JSNM | 0.501 | 0.109 | 0.911 | 0,179 | 0.699 (0.649 - 0.748) | 0.3429 |
| 14 | ∆TBT🡨∆CLIN+∆KL+JSNM | 0.500 | 0.105 | 0.911 | 0,176 | 0.679 (0.627 - 0.732) | 0.9285 |
| 15 | TBT+∆TBT🡨CLIN+KL+JSNM**^#^** | 0.534 | 0.381 | 0.917 | 0,223 | **0.739** (0.695 - 0.782) | 0.0076 |
| 16 | TBT+∆TBT🡨∆CLIN+KL+JSNM | 0.533 | 0.399 | 0.917 | 0,219 | 0.727 (0.681 - 0.772) | 0.0570 |
| 17 | TBT+∆TBT🡨CLIN+∆KL+JSNM | 0.534 | 0.393 | 0.917 | 0,222 | 0.731 (0.687 - 0.776) | 0.0224 |
| 18 | TBT+∆TBT🡨∆CLIN+∆KL+JSNM | 0.535 | 0.428 | 0.917 | 0,221 | 0.718 (0.671 - 0.765) | 0.1315 |

BACC, PPV,NPV and AP refer to balanced accuracy, positive predictive value, negative predictive value and average precision. * refers to the reference model and # refers to the retained model. NaN refers to a Not-a-Number value where the sensitivity value was equal to zero. The model with the descriptor on the left of (🡨) is adjusted for the descriptor(s) on the right of (🡨). The highest AUC value obtained is bolded. In Scenario 2, progressors were defined as knees with 1<KL<4 at baseline and with a variation of > 0 from 24 months to 48 months in JSNM only.

Table S3: Scenario 3 performance results of selected prediction models using TBT analysis of the whole tibial subchondral zone

| **Ref.** | **Model** | **BACC** | **PPV** | **NPV** | **AP** | **AUC (CI 95%)** | **pValue** |
| --- | --- | --- | --- | --- | --- | --- | --- |
| 1 |  | 0.500 | NaN | 0.870 | 0,154 | 0.569 (0.525 - 0.612) | 0.0100 |
| 2 | CLINW | 0.500 | NaN | 0.870 | NaN | 0.566 (0.523 - 0.610) | 0.0060 |
| 3 | CLIN🡨KL | 0.500 | NaN | 0.870 | 0,177 | 0.623 (0.579 - 0.666) | 0.9929 |
| 4 | CLIN🡨JSNM | 0.500 | NaN | 0.870 | 0,155 | 0.569 (0.525 - 0.614) | 0.0127 |
| 5 | CLIN🡨KL+JSNM* | 0.500 | NaN | 0.870 | 0,189 | 0.622 (0.578 - 0.667) | 1.0000 |
| 6 | TBT | 0.503 | 0.238 | 0.870 | 0,200 | 0.642 (0.601 - 0.684) | 0.4952 |
| 7 | TBT🡨CLIN+KL+JSNM | 0.506 | 0.270 | 0.871 | 0,218 | 0.670 (0.629 - 0.711) | 0.0072 |
| 8 | TBT🡨∆CLIN+KL+JSNM | 0.509 | 0.329 | 0.872 | 0,214 | 0.655 (0.613 - 0.696) | 0.1178 |
| 9 | TBT🡨CLIN+∆KL+JSNM | 0.502 | 0.187 | 0.870 | 0,202 | 0.655 (0.614 - 0.696) | 0.1931 |
| 10 | TBT🡨∆CLIN+∆KL+JSNM | 0.504 | 0.220 | 0.871 | 0,197 | 0.641 (0.599 - 0.683) | 0.4929 |
| 11 | ∆TBT🡨CLIN+KL+JSNM | 0.503 | 0.528 | 0.870 | 0,204 | 0.641 (0.596 - 0.685) | 0.2710 |
| 12 | ∆TBT🡨∆CLIN+KL+JSNM | 0.502 | 0.580 | 0.870 | 0,202 | 0.628 (0.582 - 0.674) | 0.7847 |
| 13 | ∆TBT🡨CLIN+∆KL+JSNM | 0.500 | 0.000 | 0.870 | 0,177 | 0.597 (0.552 - 0.641) | 0.3386 |
| 14 | ∆TBT🡨∆CLIN+∆KL+JSNM | 0.500 | 0.000 | 0.870 | 0,171 | 0.584 (0.538 - 0.629) | 0.1917 |
| 15 | TBT+∆TBT🡨CLIN+KL+JSNM**^#^** | 0.525 | 0.335 | 0.876 | 0,240 | **0.689** (0.649 - 0.730) | 0.0043 |
| 16 | TBT+∆TBT🡨∆CLIN+KL+JSNM | 0.528 | 0.403 | 0.876 | 0,236 | 0.675 (0.634 - 0.717) | 0.0393 |
| 17 | TBT+∆TBT🡨CLIN+∆KL+JSNM | 0.524 | 0.343 | 0.875 | 0,237 | 0.684 (0.643 - 0.724) | 0.0210 |
| 18 | TBT+∆TBT🡨∆CLIN+∆KL+JSNM | 0.525 | 0.373 | 0.876 | 0,234 | 0.671 (0.630 - 0.713) | 0.0858 |

BACC, PPV,NPV and AP refer to balanced accuracy, positive predictive value, negative predictive value and average precision. * refers to the reference model and # refers to the retained model. NaN refers to a Not-a-Number value where the sensitivity value was equal to zero. The model with the descriptor on the left of (🡨) is adjusted for the descriptor(s) on the right of (🡨). The highest AUC value obtained is bolded. In Scenario 3, progressors were defined as knees with 1<KL<4 at baseline and with a variation of > 0 from baseline to 48 months in JSNM or JSNL.

Table S4: Scenario 4 performance results of selected prediction models using TBT analysis of the whole tibial subchondral zone

| **Ref.** | **Model** | **BACC** | **PPV** | **NPV** | **AP** | **AUC (CI 95%)** | **pValue** |
| --- | --- | --- | --- | --- | --- | --- | --- |
| 1 | CLIN | 0.500 | NaN | 0.905 | 0,130 | 0.605 (0.553 - 0.656) | 0.0003 |
| 2 | CLINW | 0.500 | NaN | 0.905 | NaN | 0.599 (0.548 - 0.651) | 0.0001 |
| 3 | CLIN🡨KL | 0.500 | NaN | 0.905 | 0,145 | 0.644 (0.593 - 0.695) | 0.0031 |
| 4 | CLIN🡨JSNM | 0.500 | NaN | 0.905 | 0,161 | 0.687 (0.640 - 0.735) | 0.8603 |
| 5 | CLIN🡨KL+JSNM* | 0.500 | NaN | 0.905 | 0,161 | 0.688 (0.640 - 0.735) | 1.0000 |
| 6 | TBT | 0.504 | 0.249 | 0.905 | 0,188 | 0.679 (0.630 - 0.728) | 0.7709 |
| 7 | TBT🡨CLIN+KL+JSNM | 0.505 | 0.223 | 0.905 | 0,194 | 0.715 (0.669 - 0.760) | 0.1438 |
| 8 | TBT🡨∆CLIN+KL+JSNM | 0.507 | 0.293 | 0.906 | 0,192 | 0.699 (0.653 - 0.745) | 0.5993 |
| 9 | TBT🡨CLIN+∆KL+JSNM | 0.507 | 0.274 | 0.906 | 0,198 | 0.714 (0.667 - 0.760) | 0.1911 |
| 10 | TBT🡨∆CLIN+∆KL+JSNM | 0.508 | 0.326 | 0.906 | 0,196 | 0.697 (0.650 - 0.744) | 0.6700 |
| 11 | ∆TBT🡨CLIN+KL+JSNM | 0.501 | 0.139 | 0.905 | 0,191 | 0.707 (0.661 - 0.753) | 0.2847 |
| 12 | ∆TBT🡨∆CLIN+KL+JSNM | 0.504 | 0.499 | 0.905 | 0,187 | 0.689 (0.641 - 0.737) | 0.9654 |
| 13 | ∆TBT🡨CLIN+∆KL+JSNM | 0.501 | 0.138 | 0.905 | 0,196 | 0.707 (0.661 - 0.754) | 0.2971 |
| 14 | ∆TBT🡨∆CLIN+∆KL+JSNM | 0.505 | 0.464 | 0.906 | 0,192 | 0.687 (0.638 - 0.736) | 0.9773 |
| 15 | TBT+∆TBT🡨CLIN+KL+JSNM**^#^** | 0.542 | 0.363 | 0.912 | 0,238 | **0.744** (0.701 - 0.786) | 0.0124 |
| 16 | TBT+∆TBT🡨∆CLIN+KL+JSNM | 0.543 | 0.395 | 0.912 | 0,231 | 0.730 (0.686 - 0.774) | 0.0950 |
| 17 | TBT+∆TBT🡨CLIN+∆KL+JSNM | 0.540 | 0.353 | 0.912 | 0,234 | 0.739 (0.696 - 0.782) | 0.0250 |
| 18 | TBT+∆TBT🡨∆CLIN+∆KL+JSNM | 0.543 | 0.408 | 0.912 | 0,233 | 0.723 (0.678 - 0.768) | 0.1729 |

BACC, PPV,NPV and AP refer to balanced accuracy, positive predictive value, negative predictive value and average precision. * refers to the reference model and # refers to the retained model. NaN refers to a Not-a-Number value where the sensitivity value was equal to zero. The model with the descriptor on the left of (🡨) is adjusted for the descriptor(s) on the right of (🡨). The highest AUC value obtained is bolded. In Scenario 4, progressors were defined as knees with 1<KL<4 at baseline and with a variation of > 0 from baseline to 48 months in JSNM only.
